# Improved Alzheimer’s Disease Detection by MRI Using Multimodal Machine Learning Algorithms

**DOI:** 10.3390/diagnostics11112103

**Published:** 2021-11-13

**Authors:** Gopi Battineni, Mohmmad Amran Hossain, Nalini Chintalapudi, Enea Traini, Venkata Rao Dhulipalla, Mariappan Ramasamy, Francesco Amenta

**Affiliations:** 1 Telemedicine and Telepharmacy Centre, School of Medicinal and Health Products Sciences, University of Camerino, 62032 Camerino, Italy; mohammadamra.hossain@studenti.unicam.it (M.A.H.); nalini.chintalapudi@unicam.it (N.C.); enea.traini@unicam.it (E.T.); francesco.amenta@unicam.it (F.A.); 2 The Research Centre of the ECE Department, V.R. Siddhartha Engineering College, Vijayawada 521002, Andhra Pradesh, India; hodece@vrsiddhartha.ac.in (V.R.D.); mariappan@vrsiddhartha.ac.in (M.R.)

**Keywords:** Dementia, Alzheimer’s disease, machine learning, prediction, performance, AUROC

## Abstract

Adult-onset dementia disorders represent a challenge for modern medicine. Alzheimer’s disease (AD) represents the most diffused form of adult-onset dementias. For half a century, the diagnosis of AD was based on clinical and exclusion criteria, with an accuracy of 85%, which did not allow for a definitive diagnosis, which could only be confirmed by post-mortem evaluation. Machine learning research applied to Magnetic Resonance Imaging (MRI) techniques can contribute to a faster diagnosis of AD and may contribute to predicting the evolution of the disease. It was also possible to predict individual dementia of older adults with AD screening data and ML classifiers. To predict the AD subject status, the MRI demographic information and pre-existing conditions of the patient can help to enhance the classifier performance. In this work, we proposed a framework based on supervised learning classifiers in the dementia subject categorization as either AD or non-AD based on longitudinal brain MRI features. Six different supervised classifiers are incorporated for the classification of AD subjects and results mentioned that the gradient boosting algorithm outperforms other models with 97.58% of accuracy.

## 1. Introduction

Alzheimer’s disease (AD) is an adult-onset cognitive disorder (AOCD) which represents the sixth leading cause of mortality and the third most common disease after cardiovascular diseases and cancer [1]. AD is mainly characterized by nerve cell widespread loss, neuro-fibrillary tangles, and senile plaques occurring primarily in the hippocampus, entorhinal cortex, neocortex, and other brain regions [2]. It is hypothesized that there are 44.4 million people experiencing dementia in the world and this number will probably increase to 75.6 million in 2030 and 135.5 million in 2050 [3]. For half a century, the diagnosis of AOCD was based on clinical and exclusion criteria (neuropsychological tests, laboratory, neurological assessments, and imaging findings). The clinical criteria have an accuracy of 85% and do not allow a definitive diagnosis, which could only be confirmed by post-mortem evaluation. Clinical diagnosis has been associated with time with instrumental examinations, such as analysis of the liquoral levels of specific proteins and demonstration of cerebral atrophy with neuroimaging [4]. Further evolution of neuroimaging techniques is associated with quantitative assessment.

Various neuroimaging approaches, such as the AD neuroimaging initiative (ADNI) [4], were developed to identify early stages of dementia. The early diagnosis and possible prediction of AD progression are relevant in clinical practice. Advanced neuroimaging techniques, such as magnetic resonance imaging (MRI), have been developed and presented to identify AD-related molecular and structural biomarkers [5]. Clinical studies have shown that neuroimaging modalities such as MRI can improve diagnostic accuracy [6]. In particular, MRI can detect brain morphology abnormalities associated with mild cognitive impairment (MCI) and has been proposed to predict the shift of MCI into AD accurately at an early stage.

A further suggested approach is the analysis of the so-called multimodal biomarkers that can play a relevant role in the early diagnosis of AD. Studies of Gaubert and coworkers trained the machine learning (ML) classifier using features such as EEG, APOE4 genotype, demographic, neuropsychological, and MRI data of 304 subjects [7]. The model is trained to predict amyloid, neurodegeneration, and prodromal AD. It has been reported that EEG can predict neurodegenerative disorders and demographic and MRI data are able to predict amyloid deposition and prodromal at five years, respectively. In line with the above investigations, ML techniques were considered useful to predict AD. This helps in quick decision making [8]. Different supervised ML models were developed and tested their performance in AD classification [9]. However, it is said that boosting models [10] such as the generalized boosting model (GLM Boost) and gradient boosting machines (GBM) outperform other models in terms of classification accuracy and specificity.

Dementia can also be predicted via integrating ML knowledge with the patient’s clinical history. A gradient boosting model (light GBM) to predict the onset of dementia using two years AD patient records was proposed as well [11]. This obtained 87% of accuracy. Another approach using Recurrent Neural Networks (RNN) was presented for the AD progression modeling [12]. This network was compared with another existing RNN modeling with data assertion and regression method. This resulted in a 74% of accuracy even with unlabeled data. At the same time, MRI demographic data can also help to predict AD by learning the intradata relationships. It has been reported that with this approach random forest (RF) models outperform other classification algorithms such as SVM [13]. In particular, deep learning models produced promising results in predicting the shift of MCI into overt AD and in early AD detection [14]. Deep learning models used unlabeled data during pre-processing and are well suited for imbalanced datasets and achieving a knowledge base. It has been suggested that deep learning could be a promising solution in AD identification and symptom detection [15]. An effective and comprehensive deep learning model can help to an early AD prediction, and consequently, to provide timely treatment to the suffering patients.

Discretization of MRI data efficiently handles the outliers and thereby improves the accuracy of ML classifiers. It is reported that the successful classification of dementia subjects can be done by supervised models associated with feature selection [16]. In another study, patient classification was accomplished via multifactor affiliation analysis with the inter feature relationships [17]. This technique helps in getting better patient classification and produce higher performance compared with classification trees and generic-distribution zones [17]. The above approaches did not highlight the importance of data-centric ML techniques and the adoption of model boosting knowledge, which can transform weak learners into strong learners and improve model performance.

In this study, we have applied the datacentric ML classification techniques by involving both supervised and boosting models and comparing performance in the detection of the best model. To achieve this, we proposed an ML framework for the classification of AD and non-AD patients, and the classifier performance was assessed and validated with cross-validation techniques. This work has developed the presentation and comparison of the classification models efficiently on smaller datasets. The main purpose of this investigation was to present the list of classification accuracies along with other performance metrics, such as precision and recall. The most notable outcome for this research study is the analysis of the progression among prediction and classification of AD detection.

## 2. Methods

### 2.1. Subjects

The dataset was retrieved from the Open Access Series of Imaging Studies (OASIS) of neurology. Patients in the age group between 60 and 96 years of age were chosen from a bigger dataset of people who had taken an interest in MRI studies at Washington University. The dataset is based on the accessibility of something like two separate visits in which clinical and MRI data were recorded, three or more gained T1-weighted images per imaging session and right-hand strength. The patient database was acquired from the longitudinal pool of Alzheimer Disease Research Centre (ADRC) at Washington University [18]. The controlled group and psychologically disabled patients’ group were enlisted in the ADRC, especially through media offers, among which 80% of people by direct contact with the center and the rest of people by doctors’ referral. All the patients took part as per the rules of the Human Studies Committee, Washington University. Endorsement for public sharing of the anonymized data was also explicitly obtained. The subject demographic information is shown in Table 1.

### 2.2. Clinical Assessment

Dementia status was assessed by the Clinical Dementia Rating (CDR) scale. The classification of dementia or non-dementia control groups was based on clinical criteria, without reference to psychometric execution, and any likely reasons for dementia (known neurological, clinical, or mental issues), which would not lead to dementia. The diagnosis of AD was made based on clinical data (obtained basically from an insurance source). The subjects experienced a slow, gradual decrease in memory and other psychological and functional impairments. In particular, the CDR is a dementia scale, which rates patients for the level of impedance in every one of six areas: memory, orientation, judgment and critical thinking, work in the community, home and hobbies, and individual care. Based on the reliable source and subject meeting, the global CDR score is obtained from singular evaluations in each domain. The global CDR of 0 indicates no dementia and a CDR of 0.5, 1, 2, and 3 indicate extremely mild, mild, moderate, and severe dementia, respectively [19]. The proposed techniques here take into account the clinical finding of AD in people with a CDR of 0.5 or more prominent based on standard criteria based on histopathological assessment in 93% of the people [20]. Those in the earliest or mildest cognitive decline (CDR of 0.5) of AD might be considered as MCI. The diagnostic characteristics of different age groups considered are presented in Table 2.

### 2.3. Image Acquisition

For each subject, three or four individual T1-weighted magnetizations prepared rapid gradient-echo (MP-RAGE) images were acquired on a 1.5-T Vision scanner (Siemens, Erlangen, Germany) in a single imaging session. Head movement was minimized by padding and utilizing a thermoplastic face mask. Each image presents 14 independent features each corresponding to classify the dependent value of the subject group. The binary classifier subject group defines each individual either as non-demented (0) or demented (1). Table 3 presents the description of each independent feature allowing the classification of the subject group.

### 2.4. Experimental Setup

The experimental setup was introduced for the classification of AD patients and included

➢ A learning model that can effectively predict and segregate true AD subjects from a given population.
➢ The development of a novel ML classifier and validate its performance.

**Figure 1:**
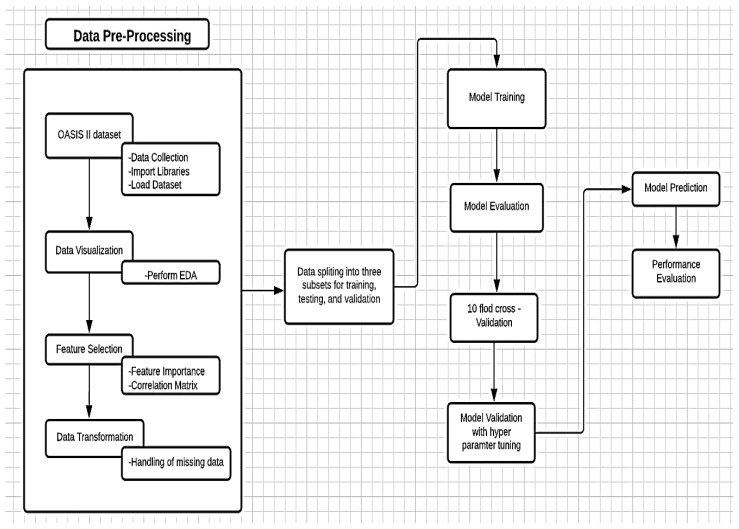
Experimental setup for the proposed model.

To achieve this, OASIS longitudinal MRI data of 150 subjects were used. The ML model pipeline approach was applied in the diagnosis of AD, to classify true dementia subjects. The proposed ML framework can learn data by the provided classifiers and categorize them as true and non-AD subjects. The Jupiter platform with Python libraries was used for an experimental setup; this platform is well known by developers for processing, assessment, and model building. Python is a high-level programming language with dynamic semantics. Figure 1 shows the proposed method to evaluate a high-performance model in AD patient classification.

#### 2.4.1. Data Pre-Processing

##### (a) Missing Data Handling

The real-world data contain missing values and noise, also in a raw format that cannot be directly involved in the development of ML models. To convert such noisy data into a machine-understandable format, data pre-processing steps are needed, such as data cleaning and data formatting. The first step in data pre-processing was the handling of missing data. In this, we identified that the SES (1–5) feature had 19 missing values and MMSE (0–30) had 2 missing values. For handling these two features, we replaced missing data points with the values that occurred the most (for SES this was 2 and for MMSE this was 30) [21].

##### (b) Data Visualization

**Figure 2:**
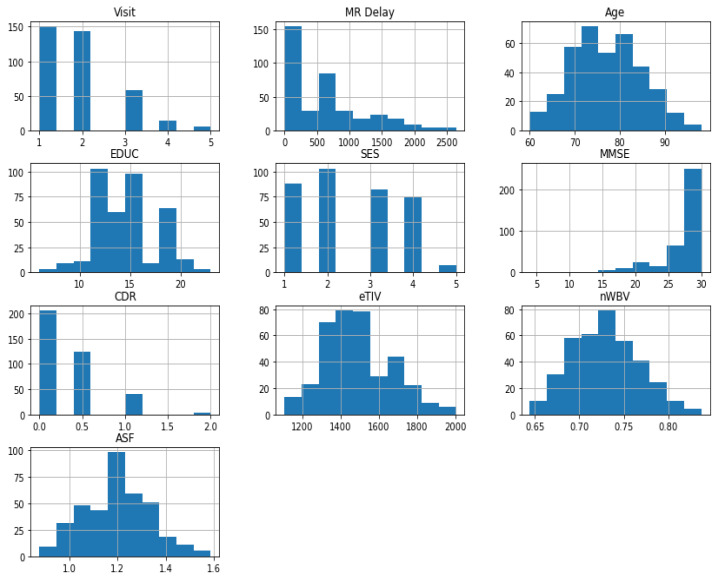
Histogram representation of the feature value distribution.

In this step, we perform an exploratory data analysis (EDA) technique that incorporates different methods and tools employed to advance the statistical insight and graphical data representation. Figure 2 represents the value distribution of different MRI features in the prediction of the target AD group value.

**Figure 3:**
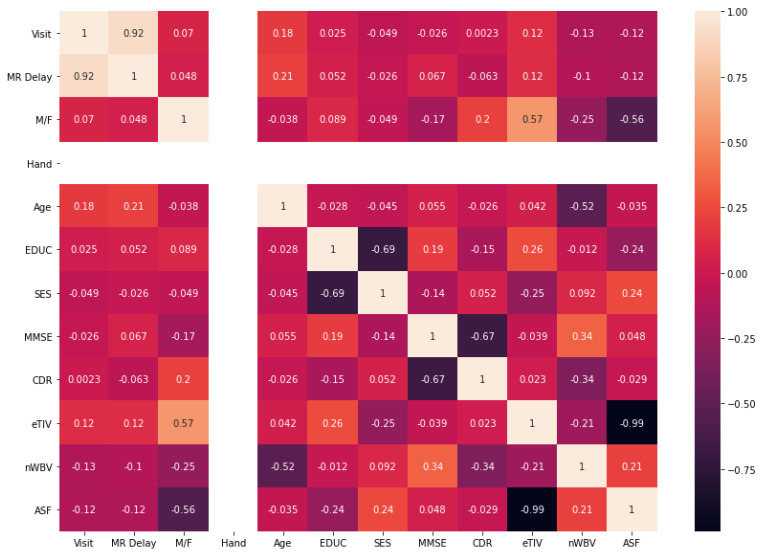
Correlation matrix heatmap after processing of missing values.

**Figure 4:**
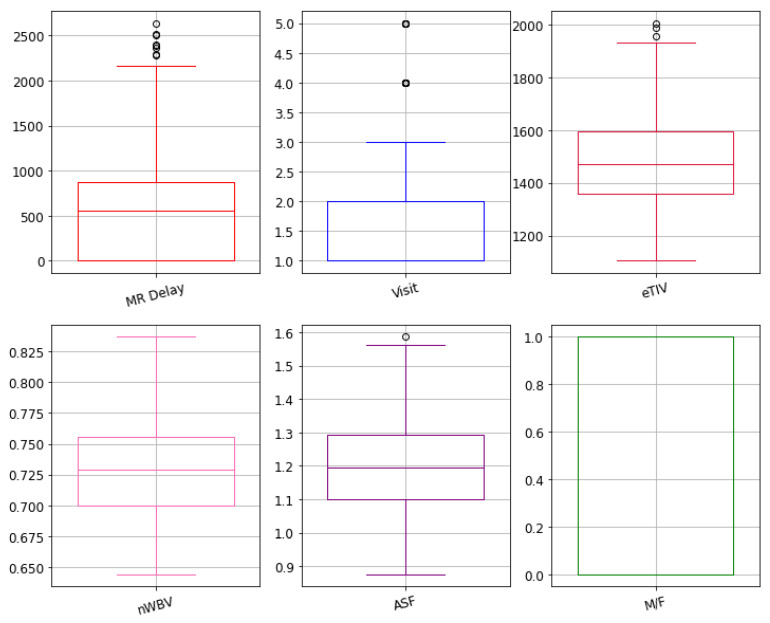
Box plot representation of the features with high correlation.

The identification of a relationship between different MRI features helps in the detection of highly correlated features with the target group. To do that correlation, a matrix was developed to understand the relationship among given features and targeted outcomes. The features with at least 50% of correlation with the target group are included. The outcome of the correlation matrix heatmap can be visualized in Figure 3. Similarly, an outlier can be a data point that varies significantly from other parameters. The dataset outliers present the quantitative distribution of data in a way that helps in the comparison of given features. The box plots of outliers with 50% of correlation were reported as e-TIV, age, n-WBV, ASF, MR delay, and VISIT are presented in Figure 4.

#### 2.4.2. Data Splitting

In this method, we divided the dataset into three subsets for cross-validation purposes. One subset is used for model prediction (i.e., test data) and the other two sets (i.e., training and validation) are used to assess model performance by training against new data. After data preprocessing, we randomly split the whole dataset into an 80:20 ratio, where 80% was used for training and 20% was used for testing. This will enable the machine to create new combinations every time to run the model and make it possible to predict it with the highest accuracy.

**Figure 5:**
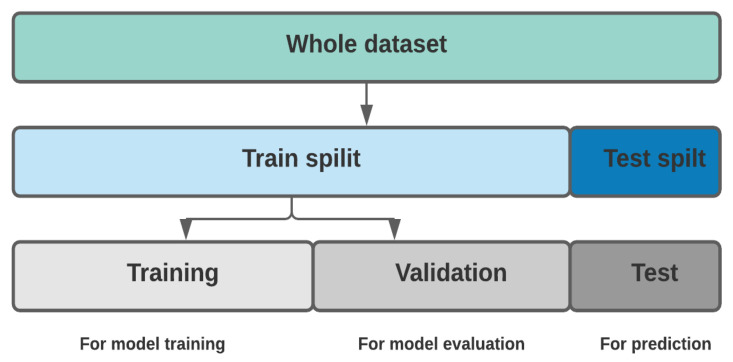
Schematic representation of the data splitting stage.

After model training, the training dataset was split into two subsets for training and validation (Figure 5). The validation dataset helps to choose hyper tuning parameters, such as regularization and learning rate. These hyper tuning parameters can limit the model overfitting and improve accuracy. After a model has been performed efficiently with a validation subset, the model stops training itself at a particular epoch to avoid repeating the same experiment.

#### 2.4.3. Training of ML Classifiers

The training of the ML classifier depends on the trained data for the prediction of the subject group across the given features. The classifier will then be well-tuned and validated on holdout data. Firstly, model training involves a process that ML can pass with the trained data and the classifier uncovers the train data patterns. Therefore, the parameters are inputted to the target variables. As mentioned, we aimed to propose an ML classifier for an explicit work of classifying AD and non-AD patients with the highest accuracy. To predict the AD patient status given to a set of independent features, we applied different supervised and ensemble learning models to propose an optimized ML classifier in AD subject categorization. Four supervised algorithms, namely Random Forest (RF), Support Vector Machines (SVM), Naive Bayes (NB), and Logistic Regression (LR), and ensemble learning models such as gradient boosting and Adaboosting, are employed to conduct model training. A brief description of each model is given below.

❖ Random Forest (RF)

The RF model is a bootstrap aggregating (bagging) model, which is implemented using a set of randomly generated decision trees or applying the divide and conquer method with random sampling, and calculates a weighted average of nodes reached [22]. For each sample taken in the training dataset, a decision tree is formed and then trained followed by grid search using 10-fold cross-validation with different parameters combinations. The classifier performance of the RF model is studied using the Gini criterion.

❖ Support Vector Machines (SVM)

The SVM is a non-linear ML classifier, which finds a hyperplane that separates the data points and classifies them into multi-dimensional space depending on the number of features [23]. It can be used for classification and regression analysis but is most often used for classification. To divide data into different classes, SVM generates the best line or decision boundary known as the hyperplane. The extreme points or vectors chosen by SVM to draw the hyperplane are known as support vectors. This hyperplane was crucial in improving the SVM model’s performance. This model is implemented initially without fine-tuning, just taking the regularization parameter, C = 1, and radial basis function as the kernel. Then, fine-tuning is done as with grid search and different combinations of ‘C’ values and kernel functions, followed by 10-fold cross-validation. Finally, its classification or prediction performance is studied with the help of a confusion matrix.

❖ Gaussian Naive Bayes (GNB)

The GNB classifier uses the Bayes theorem and is implemented using mutually independent variables [24]. An NB classifier is a probabilistic machine learning model that uses the Bayes theorem to perform classification:

$$p(A|B)=\frac{p(B|A)\;p\left(A\right)}{p\left(B\right)}$$

We calculate the probability of A occurring when features B occurred using Bayes’ Theorem. The prediction or assumption is based on a strong assumption of feature independence. The predictors or features are self-contained and unrelated to one another. Because of its predictability, this model is famous in the ML environment. The GNB model is applied as a selective classifier for dementia, which calculates the set of probabilities by counting the frequency and combination of values in a given dataset. After training the GNB model, a 10-fold cross-validation was performed.

❖ Logistic Regression (LR)

The LR classifier is a linear type that is implemented similar to the SVM with dependent and independent variables, but with a greater number of values for regularization parameter ‘C’ [25]. This model will use the ‘sigmoid function’ for the prediction probability and classifier decision boundaries.

❖ Gradient Boosting

The Gradient boosting (GB) model is an ensemble ML algorithm, which utilizes a gradient boosting structure and is built on basis of the decision tree [26]. When it is implemented for structured data, decision tree-based algorithms are performing best, whereas ensemble learning algorithms outperform other algorithms, in prediction or classification problems involving unstructured data. Here, we implement the gradient boosting machine (GBM) model to classify dementias and predict the shift of MCI to AD.

❖ AdaBoost

AdaBoosting is one of the ensemble boosting classifiers, which was proposed by Yoav Freund and Robert Schapire [27]. It is an iterative ensemble learning system, which incorporates a sequential combination of several base/weak classifiers, resulting in an efficient classifier with improved accuracy. The main concept of the AdaBoost algorithm is to set the weights of classifiers and train the sample data in each iteration to predict the unusual observations accurately with minimal error.

#### 2.4.4. Model Validation

Model validation is the practice of identifying an optimal model through skipping the train and test on the same data and helps to reduce complex overfitting issues. To overcome such an issue, we performed the cross-validation (CV) method to train the model and thereafter to calculate the accuracy [28]. It is always a challenge to validate the model with a trained dataset, and to ensure the model is noise-free, computer scientists use CV techniques. In this work, we applied the CV technique because it is a popular ML technique and produces low bias models. CV technique is also known as a k-fold approach that segregates the entire dataset into k divisions with equal size. For each iteration, the model is trained with the remaining k-1 divisions [29]. Ultimately, performance is evaluated by the mean of all k-folds for estimating the ability of the classifier problem. Usually, for the imbalanced dataset, the best value for k is 5 or 10. For this work, we applied the 10-fold CV technique, which means that model was trained and tested 10 times.

### 2.5. Performance Metrics

Once the ML model is created, the performance of each model can be defined in terms of different metrics such as accuracy, sensitivity, F1-score, and area under the receiver operating characteristic (AUROC) curve values. To do that, the confusion matrix can help to identify misclassification in tabular form. When the subject is classified as demented (1) is considered as a true positive, when it is classified as non-demented, (0) is considered a true negative. The confusion matrix representation of a given dataset is shown in Table 4.

The performance measures are defined by the confusion matrix explained below.

*Accuracy:* The percentage of the total accurately classified outcomes from the total outcomes. Mathematically, it is written as:

$$\mathrm{Acc}\;(\%)=\frac{\mathrm{TP}+\mathrm{TN}}{\mathrm{TP}+\mathrm{TN}+\mathrm{FP}+\mathrm{FN}}\times 100$$

*Precision:* This is calculated as the number of true positives divided by the sum of true positives and false positives:

$$\mathrm{Precision}=\frac{\mathrm{TP}}{\mathrm{TP}+\mathrm{FP}}$$

*Recall (Sensitivity):* This is the ratio of true positives to the sum of true positives and false negatives:

$$\mathrm{Sensitivity}\;(\%)=\frac{\mathrm{TP}}{\mathrm{TP}+\mathrm{FN}}$$

*AU-ROC:* In medical diagnosis, the classification of true positives (i.e., true demented subjects) is vital, as leaving true subjects can lead to disease severity. In such cases, accuracy is not the only metric to evaluate model performance; therefore, in most medical diagnosis procedures, an ROC tool can help to visualize binary classification.

## 3. Results

**Figure 6:**
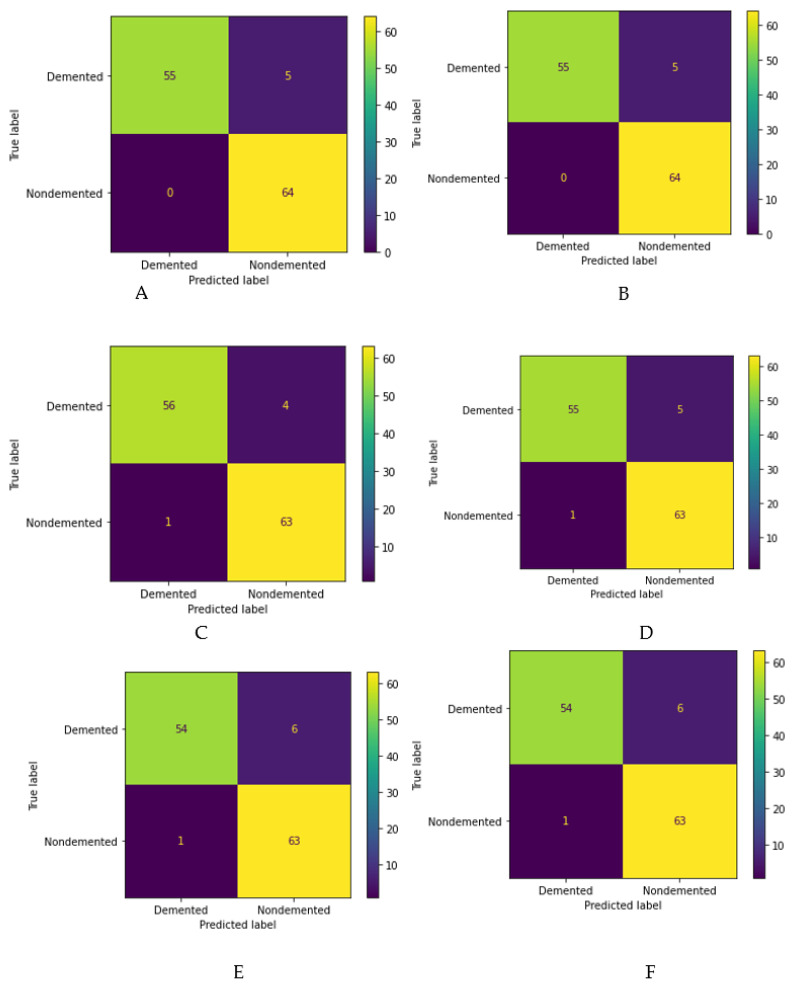
The confusion matrix outcomes of (**A**) Support vector machines (**B**) Logistic regression (**C**) Random Forest (**D**) Naïve Bayes (**E**) AdaBoosting (**F**) Gradient boosting.

After cross-validation, the classifiers were tested on a test data subset to understand how they accurately predicted the status of the AD subject. The performance of each classifier was assessed by the visualization of the confusion matrix. The confusion matrices were used to check the ML classifiers were predicting target variables correctly or not. In the confusion matrix, virtual labels present actual subjects and horizontal labels present predicted values. Figure 6 depicts the confusion matrix outcomes of six algorithms and the performance comparison of given AD classification models are presented in Table 5.

**Figure 7:**
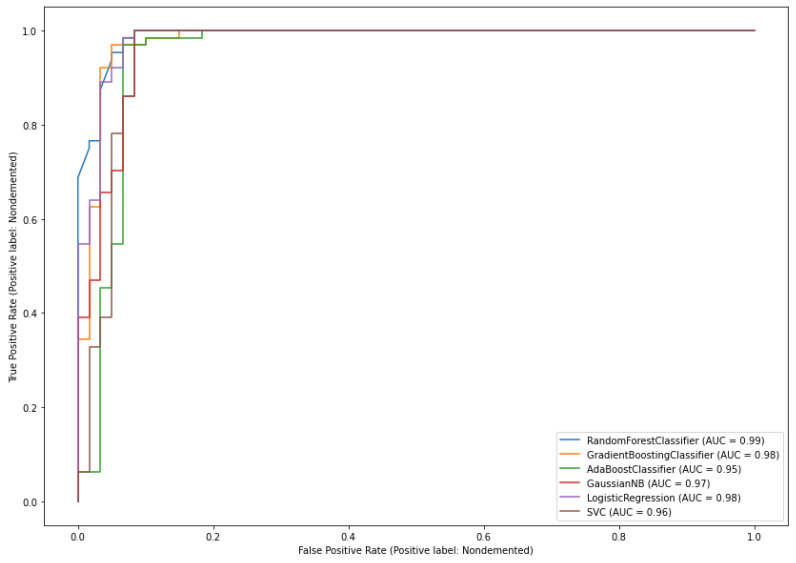
The area under the curve (AUC) of the classification performance of each algorithm.

As can be seen from Table 5, all given classifiers produced better accuracy in the classification of AD subjects, but gradient boosting outperforms all the adopted classifiers. The highest classification accuracy was achieved by the accusation of missing data with the most occurring values and features with high correlation values. It resulted in a high classification accuracy of 97.58% against 95.96% of NB classifiers with low accuracy among them. We can also observe that SVM, LR, RF, and Adaboosting have the same accuracy of 96.77%. As mentioned by [30], for imbalanced datasets, we cannot justify model performance through accuracy metrics; therefore, by creating ROC plots, conclusions can be drawn by the reliability of classification performance. Figure 7 presents the AUROC curves of the given algorithms.

The RF classifier had the highest AUC value of 0.983, which was followed by the values of gradient boosting (0.981) and NB classifier (0.980), and the lowest AUC value (0.968) was generated by SVM classifiers. LR and AdaBoosting presented AUC scores of 0.977 and 0.971, respectively. These observations indicate that boosting techniques outperformed the supervised models; in particular, the gradient boosting technique has a large capability in the classification of true AD subjects.

## 4. Discussion

Adult-onset dementia disorders have serious effects on the lifestyles of people due to the loss of cognitive functions and the progression of brain atrophy. AD is the most common form of dementia and contributes to about 60–70% of adult-onset dementia cases worldwide. Unfortunately, as already mentioned in the introduction, diagnosis of AD was based on clinical and exclusion criteria which have an accuracy of 85% and do not allow a definitive diagnosis, which could only be confirmed by post-mortem evaluation. On the other hand, an early and accurate diagnosis of AD is important for timely brain health interventions. Screening among people of AD risk in preclinical stages may prompt early identification of AD pathology and suggest better remedial procedures for complying with the AD beginning. Current biomarkers of AD have required specimen collection (such as serum or liquid) or MRI data.

Finding a computational approach for early detection of AD patients who do not exhibit any clinical signs of AD at the time of the test is an open challenge [31]. As the disease’s prevalence increases, several symptoms are found in cognitive abilities, such as language, memory, psychology, etc. Consequently, there is a need for precise and early diagnosis of dementia for helping health professionals to treat the disease at an aborning stage. There are a few techniques currently available for diagnosing adult-onset dementia. These include CSF (cerebrospinal fluid) measures, CT (Computer-based Tomography), MRI (Magnetic Resonance Imaging) assessments, ultrasound, and PET (photon outflow tomography) as a blend of neurological and psychological tests [32]. These approaches are expensive, could be partially invasive, and require time and dedicated resources. Hence, discovering effective strategies to identify dementia and finding sub-types is a challenging issue today.

Previous studies on dementia detection using conventional approaches, such as laboratory tests, patient medical history, or behavioral changes, produced less accuracy in AD diagnosis. Subsequently, computer researchers incorporated ML technologies in neurological diagnostic procedures. ML modeling was used to predict the conversion of MCI to dementia patients with a focus on cognitive reserve among 169 subjects [33]. The outcomes showed that the gradient boosting algorithm generated the highest accuracy, i.e., 93%, and also proved that cognitive reserve can play an important role in the conversion of MCI to dementia patients. It is reported that ML models can help to distinguish age-related cognitive decline (ARCD) from different dementia types (including AD, MCI, and VD) using neurocognitive tests [34].

Most health informatics researchers prefer data-centric machine learning approaches in the diagnosis of early-stage AD [35,36,37]. In data-centric approaches, data are systematically changed or preprocessed for the datasets for enhancing the performance of ML models. This is generally ignored and data collection is considered as one of the tasks. It is all about the data quality which helps to accurate data labelling [38]. The era of data-driven approaches in dementia assessment is generated with the capacity to alternate the healthcare systems with more efficiency, transparency, and personalized services for AD. The main reason behind the “dirty” AD clinical data is because there is no standardization in pathways of dementia care. For example, the dementia-related data in Northern Ireland is properly retrieved and analyzed based on the social and healthcare organizations, but the set of datasets of dementia assessments across diverse practice sites can be different. Similarly, doctors in England are also followed similar non-standardization approaches in dementia assessments [39]. This research was further validated by proposing data-driven approaches based on deep learning models from data dementia patients for calculating the agitation rates [37].

The studies with the experimental setup of ML-based data-centric methods with preprocessed MRI data can help efficient screening of MCI levels. For instance, some authors that adopted kernel density estimations for extracting texture information from the MR images and reported linear discriminant analysis (LDA) and SVM achieved high detection accuracy with limited features [40]. AD diagnosis through data preprocessing-based recursive feature elimination is proposed in [41], and results produced the highest AD subjects classification accuracy with different levels of dementia.

There is a scarcity of works that proposed data-centric ML models on demographic MRI features; rather, most of them focused on the image related datasets. Therefore, the present work strives to achieve comprehensive performance analysis in the classification of AD patients and proposed data-driven ML methodologies which utilize the data of longitudinal MRI features. Handling of missing data was done by replacing the highest occurrence value followed by normalization and standardization. With the adoption of EDA techniques, we present the feature dataset distribution and inclusion of the highest correlated features along with outliers helped us achieve the highest classification accuracy. Thereafter, we trained six different ML classifiers without reducing the dimensions of the data.

The data-driven ML classifiers were used to successfully classify the true dementia subjects and these studies were carried out by applying a combination of supervised and boosting algorithms. The advantage of conducting these types of studies can help the early identification of AD and as a result reduce medical expenses and contribute to undertaking therapeutic measures. Despite generating the highest classification accuracy, this study has some limitations, namely the small sample size involved in the final dementia subject classification. The OASIS datasets are very popular in brain studies. However, incorporation of external MRI data cannot guarantee the data quality and this can affect the study significance.

## 5. Conclusions

ML research associated with neurological studies can offer a more precise analysis of AD. We proposed a framework based on supervised learning models in the classification of AD patients into two categories, i.e., either AD or non-AD, based on longitudinal brain MRI features. It was also possible to predict individual dementia of older adults with a screening of AD data by ML classifiers. To predict the AD subject status, the MRI demographic information and pre-existing conditions of the patient can help to enhance the classification accuracy. Three classifiers (RF, NB, and Gradient boosting) produced the highest average AUC scores of 0.98. However, by considering both classification accuracy metric and AUC, the gradient boosting technique can seem a better potential classifier than others. In this study, we suggested a simple and efficient method of dementia subject identification technique by using ML classifiers. More sophisticated prediction models with detailed subject data and clinical features around the world should be investigated in future studies.

## Figures and Tables

**Table 1:** Demographic characteristics of the subjects investigated (D: demented; ND: nondemented; SD: standard deviation).

Subjects	78 D	72 ND
Male	40 D	22 ND
Female	38 D	50 ND
Age range (years)	60–96	
Median	77.0	
Mean ± SD	77.01 ± 7.3	

**Table 2:** Age and characteristics of the individuals investigated on the first clinical visit [18].

		Non-Demented					Demented				
Age	N	n	Mean	Male	Female	Convert	n	Mean	Male	Female	CDR (0.5/1)
**60s**	34	23	65.71	6	17	3	11	65.67	8	3	8/3
**70s**	71	35	74.91	11	24	4	36	73.97	20	16	29/7
**80s**	41	26	84.30	9	17	7	15	82.33	7	8	13/2
**90s**	4	2	92.50	0	2	0	2	93.00	1	1	1/1
**Total**	150	86	75.82	26	59	14	64	74.95	36	29	52/13

**Table 3:** Dataset feature description.

Features	Description
Subject ID	Subject identification number
MRI ID	Image identification number of an individual subject
Visit	Number of subject visits
Gender	Male/Female
Hand	Right/Left-handed
EDUC	Subject education level (in years)
SES	Socioeconomic status
MMSE	Mini-mental state examination score
CDR	Clinical dementia rating score
e-TIV	Estimated total intracranial volume result
n-WBV	Normalized whole brain volume result
ASF	Atlas scaling factor
Age	Subject age while scanning
Group	Demented/Nondemented/Converted
MR delay	Magnetic resonance (MR) delay is the delay time that is before the image procurement

**Table 4:** Confusion matrix of demented subjects.

Classification	1	0
D = 1	TP	FN
ND = 0	FP	TN

D: demented; ND: nondemented; TP: true-positive; TN: true-negative; FP: false-positive; FN: false-negative.

**Table 5:** Performance results of binary classification of each classifier.

N	Classifier	Accuracy	Precision	Recall	F-Score	AUROC
1.	Gradient boosting	97.58	0.98	0.96	0.97	0.981
2.	SVM	96.77	0.98	0.95	0.96	0.968
3.	LR	96.77	0.98	0.95	0.96	0.977
4.	RF	96.77	0.96	0.96	0.96	0.983
5.	AdaBoosting	96.77	0.96	0.96	0.96	0.971
6.	NB	95.96	0.96	0.95	0.95	0.980

## Data Availability

Not applicable.
